# On the role of residue phosphorylation in 14-3-3 partners: AANAT as a case study

**DOI:** 10.1038/srep46114

**Published:** 2017-04-07

**Authors:** Diego Masone, Marina Uhart, Diego M. Bustos

**Affiliations:** 1 Instituto de Histología y Embriología (IHEM) - Consejo Nacional de Investigaciones Científicas y Técnicas (CONICET), CC56, Universidad Nacional de Cuyo (UNCuyo), 5500, Mendoza, Argentina; 2 Facultad de Ingeniería, Universidad Nacional de Cuyo (UNCuyo), Mendoza, Argentina; 3 Facultad de Ciencias Exactas y Naturales, Universidad Nacional de Cuyo (UNCuyo), Mendoza, Argentina

## Abstract

Twenty years ago, a novel concept in protein structural biology was discovered: the intrinsically disordered regions (IDRs). These regions remain largely unstructured under native conditions and the more are studied, more properties are attributed to them. Possibly, one of the most important is their ability to conform a new type of protein-protein interaction. Besides the classical domain-to-domain interactions, IDRs follow a ‘fly-casting’ model including ‘induced folding’. Unfortunately, it is only possible to experimentally explore initial and final states. However, the complete movie of conformational changes of protein regions and their characterization can be addressed by *in silico* experiments. Here, we simulate the binding of two proteins to describe how the phosphorylation of a single residue modulates the entire process. 14-3-3 protein family is considered a master regulator of phosphorylated proteins and from a modern point-of-view, protein phosphorylation is a three component system, with writers (kinases), erasers (phosphatases) and readers. This later biological role is attributed to the 14-3-3 protein family. Our molecular dynamics results show that phosphorylation of the key residue Thr31 in a partner of 14-3-3, the aralkylamine N-acetyltransferase, releases the fly-casting mechanism during binding. On the other hand, the non-phosphorylation of the same residue traps the proteins, systematically and repeatedly driving the simulations into wrong protein-protein conformations.

The biological role of 14-3-3 protein family is to regulate phosphorylated proteins. The heart of cell signaling by this post-translational modification is the switching of proteins between inactive and active states, and back on. A proposed biological role attributed to 14-3-3 protein family is to be a reader, meaning that the protein binds the modified partner. However, it has been hypothesized that 14-3-3s may also accelerate the phosphorylation process acting as scaffold proteins. 14-3-3 proteins use the consensus binding motifs RSXpS/TXP (mode 1), RXY/FXpS/TXP (mode 2) and pS/TX_1−2_-COOH (mode 3), where X represents any amino acid, pS/T phospho-serine or threonine and -COOH stands for the C-terminus, to generate the high-affinity native complexes with their partners^1^.

More than 2000 proteins were experimentally tested to interact with one or more paralogs of 14-3-3, but theoretically each phosphorylated protein in serine or threonine residues could be a substrate of this family. Although the components of the system are known, the mechanisms by which 14-3-3 proteins recognize so many different partners remain unclear. An approximation came from the fact that almost all 14-3-3 partners are or contain intrinsically disordered proteins or regions (IDRs)^1^. The first proposed model of molecular recognition involving disordered regions predicted binding with high specificity, low affinity, and under thermodynamic control^2^. An example is the binding of C-terminus of the plant plasma membrane H^+^-ATPase to 14-3-3c^3^. Notably, peptide binding to 14-3-3 is an enthalpy-driven 
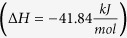
 and entropically unfavorable 
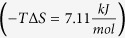
 process. This information masks specific functions of structurally constrained residues in the globular region of disordered proteins, which contribute to the stabilization of intermediate states, showing that a phosphorylated residue is necessary but not enough for the binding to 14-3-3 proteins^4^. It has been theoretically hypothesized that in the ‘fly-casting’ model, disordered proteins bind weakly and non-specifically to their targets and fold as they approach to the cognate binding site^5^. In a broad sense this model could be interpreted in two steps: first, a conformational search (changes take place before association) and second, an induced fit, where changes take place in the receptor site.

The amazing increase in computational power together with the improvements of force-fields^6^,^7^,^8^ made it possible for molecular dynamics simulations to accurately describe fine atomistic details of protein-protein complexes^9^,^10^. Limited computer power has historically been a problem for classical molecular dynamics to obtain realistic information of protein interactions. However, the development of alternative methods in which sampling is enhanced by additional bias potentials applied to a selection of degrees of freedom (collective variables), has enormously increased molecular dynamics possibilities^11^,^12^.

Here, we rely on a combination of restrained and unrestrained molecular dynamics together with experimental data to study the problem of protein-protein complex formation of 14-3-3*ζ* and its IDR partner aralkylamine N-acetyltransferase (AANAT). In an attempt to understand how phosphorylation modulates disordered states of proteins, we studied the conformational search of the disordered region of AANAT before and after phosphorylation at atomic resolution and with full length proteins. The strategy stands on the association of experimental information with specifically designed collective variables, to perform restrained dynamics with limited degrees of freedom in this protein-protein system.

## Results

### Molecular recognition. AANAT perspective

**Figure 1:**
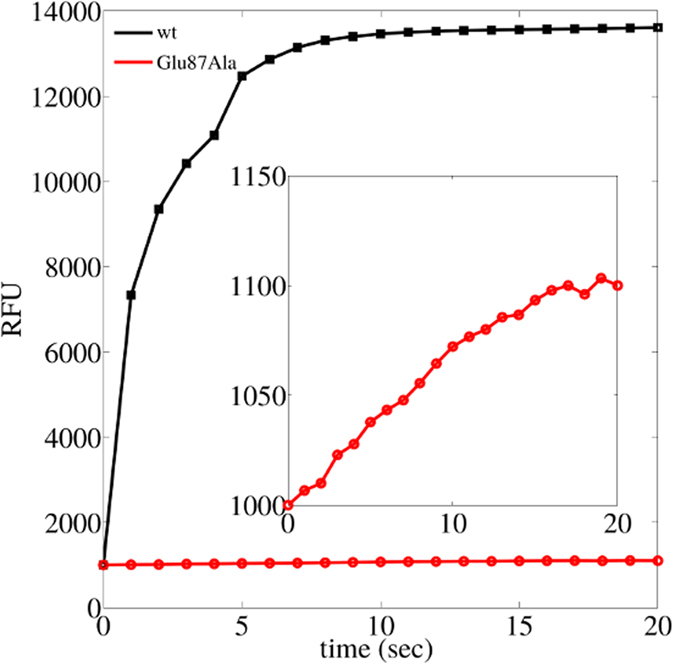
Stopped-flow polarized fluorescence experiment. Here, wt and Glu87Ala AANAT where phosphorylated and the binding to 14-3-3*ζ* was studied. RFU stands for relative fluorescent units.

Following phosphorylation of Thr31 in AANAT, 14-3-3*ζ* stimulates the production of melatonin by protecting ANAAT from the proteasome degradation^13^. In the final complex, 14-3-3*ζ* binds directly to AANAT through three H-bonds between phosphorylated Thr31 and three absolutely conserved amino acids in 14-3-3*ζ* (Arg127, Arg56, Lys49). Free AANAT contains IDRs, but after binding, these regions match the major binding groove of 14-3-3*ζ* formed principally by *α*-helices 4 and 5. To identify possible conformational changes happening during binding, we performed a stopped-flow experiment with the wt proteins and the Glu87Ala AANAT mutant, previously identified by us^4^. We already postulated on theoretical basis and also demonstrated experimentally through *in vitro* pull down and *in vivo* time course evaluation of recombinant WT and Glu87Ala AANAT mutant that this residue (Glu87) is important during binding. Those were all final-point experiments, in which the formation of the final complex was evaluated. Here, we perform a stopped-flow polarized fluorescent experiment (Fig. 1) to evaluate the effect of the Glu87Ala AANAT mutant on the binding kinetics, and to identify if the mutant has less affinity or does not bind to 14-3-3 at all. In this case wt and Glu87Ala AANAT mutant where phosphorylated in the Thr31 and binding to 14-3-3*ζ* was analyzed. The result of this stopped-flow experiment corroborates and extends our previous experimental and computational analysis^4^. Although AANAT Thr31 is not involved in the final complex (which can be readily observed in the complex crystallographic structure, PDB ID: 1IB1), this current experiment suggests that it does participate specifically in the brief pseudo native intermediate (see Fig. 1 and discussion).

Specific residue phosphorylation of 14-3-3 partners (Thr31 in AANAT) is required for high-affinity final native complex formation. However, amino acids outside the binding motif are necessary to make the first contacts between the proteins and to generate a pseudo-native complex from where the conformational search to the final native complex begins. To elucidate the mechanism by which AANAT and 14-3-3*ζ* bind, we used a protocol including unrestrained and harmonically restrained molecular dynamics. These experiments allowed us to identify weakly populated intermediate states, and to provide insights into their structure, population, and molecular mechanisms. Although the phosphorylation effect in proteins is manifold, it remains largely unexplored whether it can regulate conformational changes of proteins, and which are the effects on molecular recognition.

**Figure 2:**
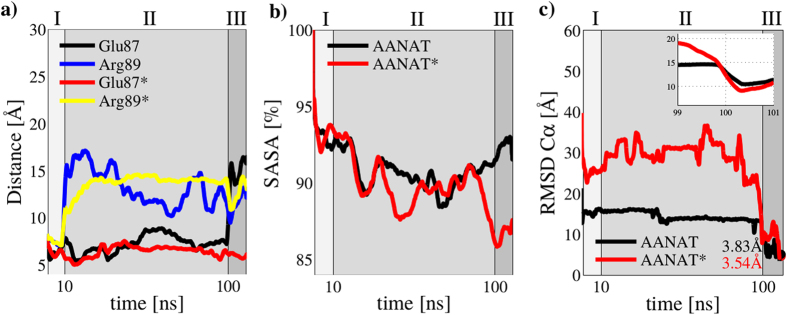
Full molecular dynamics process from initial structures, through pseudo-native to native complex. (**a**) Glu87 and Arg89 distance to groups 1 and 2 in 14-3-3*ζ* (see the methods section). The asterisk means simulation was performed with Thr31 in AANAT in its phosphorylated state (**b**) Normalized solvent accessible surface area (SASA) of the full protein-protein complex. (**c**) RMSD C*α* to native structure. Sections marked as I, II or III in the plots correspond to first harmonic potential applied (I), free simulation (II) and second harmonic potential applied (III).

**Figure 3:**
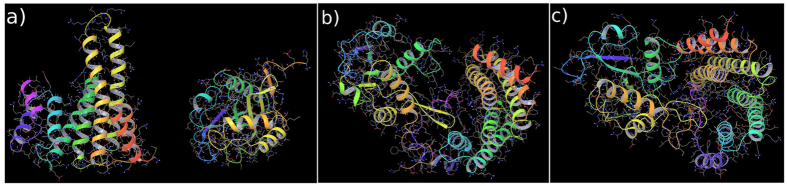
Molecular dynamics snapshots. (**a**) Initial system, 14-3-3*ζ* (left) and AANAT (right) are 60 Å apart (t = 0 ns). (**b**) Pseudo-native complex (t ~ 10 ns) after applying the first harmonic potential. (**c**) Final complex (t = 110 ns) with Root Mean Square Deviations RMSD(C*α*) = 3.53 Å, after applying the second harmonic potential.

In this work, we compared results from unphosphorylated and phosphorylated AANAT during binding to 14-3-3*ζ* protein. As we previously described, there are ‘anchor’ amino acids in the globular part of AANAT that make the first contact to 14-3-3*ζ*. Their mutation to Ala impairs binding even if AANAT is phosphorylated^4^ (see Fig. 1). In this previous study^4^, we theoretically proposed that anchor amino acids provide steric constraints that help to stabilize a native-like intermediate. However, this was never experimentally proved. With this information we performed restrained dynamics for 10 ns of simulation time (Fig. 2 region I) applying a harmonic potential between anchor residues in AANAT and their partners in 14-3-3*ζ* (see details in the methods section). In this way we forced the formation of a pseudo-native complex. After that, we performed 90 ns of unrestrained dynamics (Fig. 2 region II) allowing for the system to freely evolve. In the final region (Fig. 2 region III), we applied another harmonic potential to guide the proteins to their x-ray solved structure (PDB ID: 1IB1). Figure 3 shows representative snapshots of the three different regions in our simulations, from the initial system where proteins are at 60 Å apart, to the final complex at only RMSD(*Cα*) = 3.53 Å to the crystallized structure.

From these calculations we obtained key information that describes complex formation in two situations: with Thr31 in its phosphorylated state (AANAT*) and in its non-phosphorylated one (AANAT) (see Fig. 2). As previously pointed out^4^, Glu87 and Arg89 are the anchor residues in AANAT and they are necessary to form the pseudo-native complex. As shown in Fig. 2, in the first 10 ns (region I), when the system is forced to approximate to the pseudo-native complex, Glu87 and Arg89 follow similar trajectories in the simulations with and without phosphorylation of Thr31 in AANAT protein. In the second region (from 10 ns to 100 ns) where no harmonic potential was applied, Arg89 of AANAT differs from AANAT*, exploring a much different conformational space. Finally, in the third and last region, Arg89 follows similar paths either if the phosphoryl group is or is not present in Thr31. In contrast, it seems that Glu87 follows a similar path in both simulations during unrestrained dynamics, however, in the last region with the second harmonic potential applied, it is noticeable that Glu87 locates in a wrong position if the phosphoryl group is absent in Thr31, possibly in the opposite side of the complex interface.

Evaluation of the solvent accessible surface area (SASA) shows similar values along ~70% of the total simulation time (for both AANAT and AANAT*). After that, phosphorylated AANAT finds the right conformations and SASA starts to decrease quickly towards values similar to those observed in the crystal structure (SASA crystal = 81.02%). At the same time, non-phosphorylated AANAT is unable to adopt equivalent conformations as AANAT* making SASA values to increase (see Fig. 2b). Although both simulations ended with complexes at RMSD(*Cα*) ≤ 5 Å (Fig. 2c) it is remarkable that these structures are considerably different from each other in SASA terms.

### Molecular recognition: the fly-casting model

**Figure 4:**
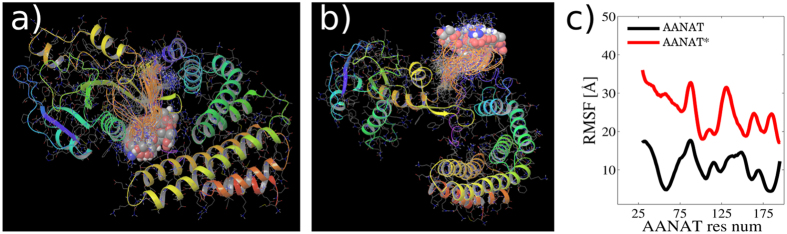
The fly-casting mechanism. (**a**,**b**) Superposition of molecular dynamics snapshots during 10 *ns* ≤ *t* ≤ 100 *ns*. Thr31 is highlithed in vdW representation. (**a**) non-phosphorylated Thr31. (**b**) phosphorylated Thr31. (**c**) Root Mean Square Fluctuations (RMSF) for phosphorylated and non-phosphorylated AANAT during 10 *ns* ≤ *t* ≤ 100 *ns*.

Fly-casting conformational space exploration was proposed by Wolynes and collaborators as a mechanism for speeding up molecular recognition when one of the partners is intrinsically disordered^5^. A clear evidence of this mechanism is observed in Fig. 2c This figure shows that phosphorylated AANAT explores larger distances and with more fluctuations (larger deviations, Fig. 2c) than non-phosphorylated AANAT, that is almost flat and much smaller. This demonstrates that AANAT* explores a larger conformational space than AANAT. Figure 4a,b picture two clusters of comparable snapshots from the unrestrained region of the simulation of AANAT and AANAT*. It is observed that Thr31 is kept trapped in the protein-protein interface when the phosphoryl group is absent (Fig. 4a), whereas in the other simulation the N-terminal region of AANAT* is outside the protein-protein interphase and increase its radius of gyration (see Fig. 7). Also, Fig. 4c shows that the fly-casting exploration includes the first ~25 amino acids of AANAT*, that behave here as a fishing net from residues 30 to 57, containing the binding motif to 14-3-3*ζ*, which includes phosphorylated Thr31.

**Figure 5:**
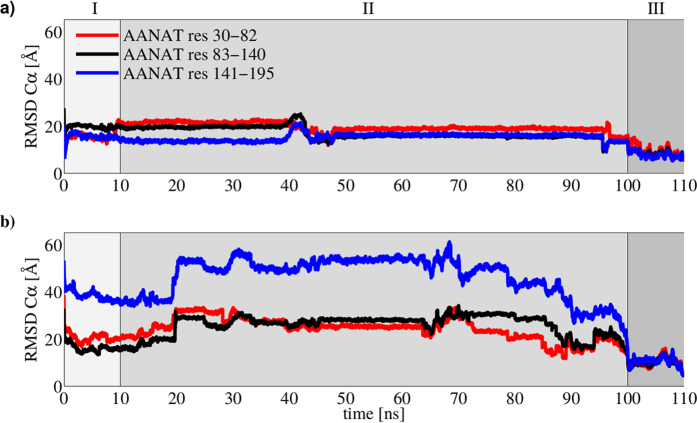
RMSD for non-phosphorylated AANAT (**a**) and phosphorylated AANAT (**b**). The protein was segmented in three regions.

Remarkably, AANAT residues from 141 to 198 exhibit a very different behavior when Thr31 is or is not phosphorylated. In Fig. 5, high frequency fluctuations of the RMSD C*α* when Thr31 is phosphorylated are compared to the almost flat-like curves for the non-phosphorylated state. This behavior suggests long-range interactions between Thr31 and C-term in AANAT. In particular, when Thr31 is phosphorylated, residues in the other side of the protein (namely, residues 141-198) begin to find their near-native geometry after major conformational fluctuations with a clear systematic lowering of their RMSD after t = 70 ns. In contrast, the same residues for non-phosphorylated Thr31 AANAT remain unable to explore the conformational space, maintaining a much lower RMSD value (near to 20 Å). All RMSD values were measured towards the crystallographic structure AANAT/14-3-3*ζ* (PDB ID: 1IB1).

### Molecular recognition: 14-3-3 perspective

**Figure 6:**
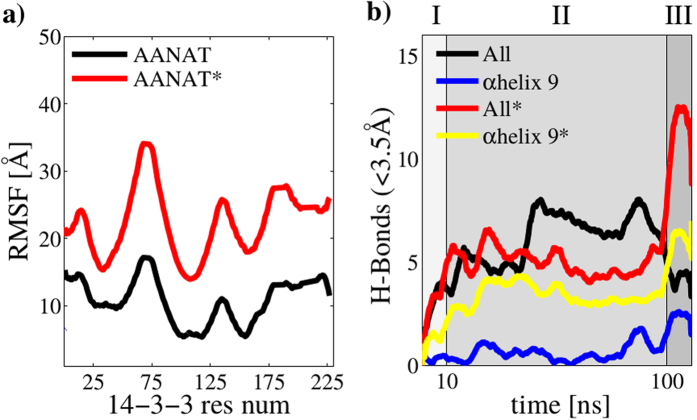
Molecular recognition. (**a**) RMSF for phosphorylated Thr31 in AANAT and non-phosphorylated Thr31 in AANAT during 10 *ns* ≤ *t* ≤ 100 *ns*. (**b**) H-bond count between proteins using a 0.35 nm cutoff for the full protein-protein system and only residues belonging to *α*helix 9 (positions from 200 to 228 in 14-3-3*ζ*).

14-3-3 protein family is considered a master regulator of phosphorylated proteins, binding more than 2000 different clients in a many-to-one, disordered-to-structured fashion. Figure 6 shows the fluctuation of 14-3-3*ζ* during both simulations (AANAT, AANAT*). Our results indicate that the binding pocket is the most mobile region of 14-3-3. The proposed auto-inhibitory C-terminal of 14-3-3 that contains the *α*-helix 9 is flexible, however, its importance is more clearly observed when we analyzed H-bonds. Remarkably, the phosphorylation of Thr31 in AANAT* produced an increase in the number of H-bonds towards *α*-helix 9 comparable to the numbers of H-bonds between AANAT and the entire 14-3-3*ζ* protein. Although the total number of H-bonds in region II (unrestrained simulation) are independent of the phosphoryl group in Thr31 (comparing the black line with the red one in Fig. 6b), the phosphorylation causes a high increase in region III. Also, almost all H-bonds of phosphorylated Thr31 are formed towards *α*-helix 9 in 14-3-3*ζ* (comparing the yellow line with the red one in the Fig. 6b). Taken together, this information could suggest that *α*-helix 9 is the main driving force to promote the right conformation and location of phosphorylated Thr31 in the major binding groove of 14-3-3*ζ*.

### Integration AANAT/14-3-3

**Figure 7:**
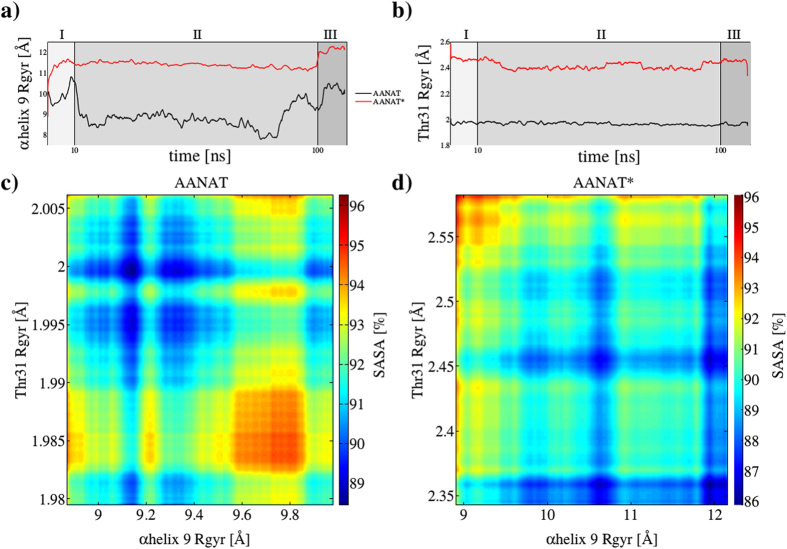
Capture radius analysis. (**a**) *α*-helix 9 (residues from 200 to 228 in 14-3-3*ζ*) shows significantly larger movements when Thr31 is phosphorylated (red line) versus unmodified Thr31 (black line). (**b**) Comparative displacement of modified (red line) and unmodified (black line) Thr31. (**c**) Surface heat map of capture radius of unphosphorylated Thr31, *α*-helix 9 and normalized SASA (**d**) The same as (**c**) but with phosphorylated Thr31.

Our results show that Thr31 phosphorylation has broad implications in the binding process, contributing not only to stabilize the final complex. To visualize this, we calculated the capture radius of phosphorylated Thr31 and *α*-helix 9 together with the complex compactness or nativeness represented by its SASA^5^,^14^. Figure 7 shows an analysis of the capture radius of the two main structural components in the binding process, AANAT Thr31 and 14-3-3*ζ α*-helix 9. Figure 7a,b show that when Thr31 is phosphorylated (red line) capture radius increases significantly. Observing SASA, it is noted that the minimum is higher in the non-phosphorylated than in the phosphorylated situation (88.5% versus 86%). Additionally, the minimum SASA observed in AANAT* corresponds to the end of region III, when the harmonic restraint has been applied for 10 ns, conducting the complex towards its final crystallized form. However, in AANAT the minimum SASA is observed during the exploration region II. Noticeably, at the end of region III, even when the final structure satisfies the restrictions imposed by the harmonic potential, it is evident that the complex is not optimized.

## Discussion

Binding between intrinsically disordered and ordered proteins is an important field and molecular dynamics simulations is one of the most suitable methods to study it, especially to answer mechanistic questions. We have described conformational changes of an intrinsically disordered protein, AANAT, before and after its phosphorylation and during the binding to 14-3-3*ζ*, an ordered scaffold protein. For statistical robustness, we run three independent experiments giving equivalent results and conclusions (each one including three 110 ns simulations with and without Thr31 phosphorylation, summing up to total simulation time of more than 300 ns × 2). Full details and plots of all quantities measured in each simulation run are included in the section Supplementary Information. In a previous work, we have theoretically proposed the presence of a metastable pseudo-native complex between these two proteins, and described the role of anchor residues in the process. Glu87 contributes to the binding process between AANAT and 14-3-3*ζ* via specific intermolecular contacts, and a loss of affinity is observed when mutating this specific residue to Ala (Fig. 1). However, our stopped-flow experiment showed no indications of less affinity complexes during binding of Glu87Ala mutant AANAT and 14-3-3*ζ*, therefore the dynamics of this process towards the final stable complex remained unexplained.

Some articles were published describing the binding mechanisms using the model system KID domain of the transcription factor CREB by free molecular dynamics. However, for the time being, equilibrium all-atom molecular dynamics simulations of coupled folding and binding are out of reach for the majority of researchers. It is clear then that the best opportunity for obtaining atomic-resolution information on coupled folding and binding relies on the use of enhanced sampling methods^15^, like harmonically restrained potentials. KID phosphorylation induces a minor shift in the equilibrium distribution of folded regions. Binding of kinetically locked and constrained regions can give other transient interactions time to form, potentially rising the number of productive binding events and thus increasing the affinity for the binding partner. Similarly, kinetically locked regions that participate in molecular recognition could frustrate the unbinding process. This would result in a shift of the binding equilibrium towards the bound state^16^,^17^. Another example is the phosphorylation of KH1 domain of KSR, which unfolds and creates a site for 14-3-3*ζ* binding^18^. KH1 domain interacts with 14-3-3*ζ* only when it is phosphorylated and unfolded. AKT phosphorylation of KH1 is responsible for its unfolding, which forms the binding site for 14-3-3*ζ*. Structural rearrangement upon phosphorylation is a common regulatory mechanism^19^, but there are no reported examples, to the best of our knowledge, of a complete protein-protein binding analysis.

Our results indicate that phosphorylation on 14-3-3*ζ* target proteins has broad implications during the binding process and not only in the stabilization of final complexes. AANAT has two canonical sites, one in the N-terminal and the other in the C-terminal of the protein. However, the complex crystal structure can only be solved when the second C-terminal binding site (considered of lower affinity) has been removed^46^. Here, we used the elementary unit (one monomer of 14-3-3*ζ* and C-terminal truncated AANAT) to mechanistically analyze how phosphorylation affects binding between AANAT and 14-3-3*ζ*. In order to reduce computational costs, we applied a protocol divided in three regions with a total of 110 ns of simulation time. For statistical robustness, we run three independent simulations giving identical results and conclusions. In the first and last 10 ns, we applied a harmonic potential to induce the formation of the pseudo-native and native complexes, respectively. In region II, we run 90 ns of unrestrained dynamics. We clearly observed that phosphorylation of Thr31 gives this residue (and the surrounding amino acids) the ability to explore more conformations following a mechanism called ‘fly-casting’^5^. During this process, the capture radius is increased and the binding process enhanced. We calculated the capture radius as the radius of gyration of the residues of interest (see equation 2) and observed a 40% increase for Thr31 (see Fig. 7b). Surprisingly, this phosphorylation has also implications in 14-3-3*ζ*, mainly through long-range interactions. We observed that the major regulatory region on 14-3-3 (*α*-helix 9) is also affected by Thr31 phosphorylation in AANAT as the movement of this region (*α*-helix 9) is also increased (Fig. 7a). These enhanced movements allow the protein-protein system to reach a more compact final complex at the end of our simulations. To measure this, 3D-SASA maps are used here as semi-quantitative estimates of the desolvation free energy upon protein complexation^20^. For our two conditions (AANAT and AANAT*) the final complex is ~3.5 Å with respect to the complex crystal structure, however, in the AANAT situation the final structure has much more solvent exposed surface than in the AANAT* one, suggesting a less stable conformation (see Fig. 7c,d).

The initial association between proteins containing IDR is often strongly dependent on ionic strength, demonstrating that specific or non-specific charge-charge interactions govern the rate of association^21^. The electrostatic field guides the protein to its target and thus accelerates the binding rate. Protein flexibility also facilitates binding via the fly-casting mechanism^22^. Our results strongly agree with this interpretation. 14-3-3*ζ* is a protein with a net negative charge^23^ and a positive hot spot where the phosphoryl group of its partners docks, demonstrating that phosphorylation of Thr31 on AANAT is the key through the binding process.

The current vision of protein phosphorylation has a question with no evident answer: is single residue phosphorylation mediated by a scaffold protein? and in particular, are 14-3-3 proteins only readers of the modification? As it has been shown in this work, phosphorylation has effects on both the partner and the reader protein in a wide sense. We propose that phosphorylation must occur before protein-protein binding and that 14-3-3 proteins only read the modification and do not participate in it.

## Methods

### Molecular dynamics

Computational experiments were independently run three times. All simulations were performed with Gromacs-5.0.4^24^,^25^ patched with Plumed 2.2.1^26^ under the GROMOS 54a7 force field, which has been extensively used for protein systems^27^,^28^. GROMOS successive and recurrent parameterizations over the recent and past years, have contributed for better protein simulations, with more reliable results and achieving better agreement with experimental data^29^,^30^,^31^,^32^. In all cases a time step of 2 fs was used with all bond-lengths constrained using the sixth-order LINear Constraint Solver (LINCS) algorithm^33^. Systems were solvated in TIP3 water model, minimized with the steepest descent method and equilibrated for 5 ns under the NVT ensemble using Nose-Hoover’s^34^ thermostat coupling with time constants set to 1 ps. Production MD runs where performed for a total of 110 ns under the NPT ensemble using Nose-Hoover’s^34^ thermostat and Parrinello-Rahman’s barostat^35^ at 303.15 K^36^,^37^,^38^ with periodic boundary conditions in all directions. The reference pressure was set to 1 bar under isotropic coupling conditions, time constants for the thermostat and the barostat were set to 1 ps and 5 ps, respectively and compressibility was set to 4.5*x*10^−5^ *bar*^−1^. Particle-Mesh-Ewald (PME) method was used for long-range electrostatics^39^. Crystal structures of 14-3-3 protein *ζ* isoform and Serotonin *N*-acetyltransferase (AANAT) are available in the Protein Data Bank (PDB IDs: 1A4O^40^ and 1L0C^41^ respectively). Missing residues 160-166 for 14-3-3 in PDB ID: 1A4O were predicted with a loop prediction methodology using the freely available web-server FREAD^42^. Systems were prepared for molecular dynamics using the also freely available web-server CHARMM-GUI^43^,^44^,^45^.

### Pseudo-native complex

Starting from the individually crystallized monomers at t = 0 ns (Fig. 3a) we induced the pseudo-native complex formation between proteins 14-3-3 and AANAT through restrained molecular dynamics using a reaction coordinate that imposed a 5 Å distance condition between 14-3-3 and AANAT selected residues (AANAT Glu87 with 14-3-3 group1: Lys49, Arg56, Arg127, Asn173 and AANAT Arg89 with 14-3-3 group2: His164, Pro165, Ile166, see the pair of equations 1). The reaction coordinate was defined to be applied on the center of masses (COM) of the residues of interest.


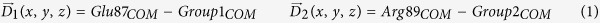


### Final complex

To induce final near-native complex formation, we used again distance constraints taken from the crystal structure of the 14-3-3*ζ*/AANAT complex, available in the Protein Data Bank (PDB ID: 1IB1)^46^. Selected residues are indicated in Table 1 together with the distance condition imposed to their centers of masses (as measured from the complex crystal sctructure). In all cases, before computing RMSF(*Cα*) for AANAT and 14-3-3 proteins at each trajectory step, a *Cα* least squares superposition to the reference crystallographic complex (PDB ID: 1IB1) was performed.

The complete simulation process is highlighted as three different regions in all figures plotting time (0–110 ns). Region I (0–10 ns) corresponds to the pseudo-native complex formation by forcing the system to reach the target distances 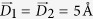. Region II (10–90 ns) is unrestrained molecular dynamics to let the system relax before a newer condition is imposed. Region III (100–110 ns) belongs to final near native complex formation using 6 simultaneous conditions, as listed in Table 1. To take into account for the effect of Thr31 phosphorylation in the protein-protein recognition process, the complete simulation procedure was repeated with phosphorylated Thr31. To ensure statistical relevance, all simulations (AANAT and AANAT*) were repeated twice, for a total amount of more than 600 ns.

Radius of gyration was used here as a measure of space conformational exploration and was calculated using the default implementation available in Plumed (see equation 2, where *r*_*COM*_ is the position of the center of mass of the involved residues and *r*_*i*_ and *m*_*i*_ are respectively the position and mass of each atom belonging to the *n* selected residues, defined by the sum running from i to n.)


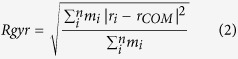


To estimate interaction free energies and to calculate the contribution per residue we used the g_mmgbsa tool^47^, which follows a Molecular Mechanics Poisson-Boltzmann surface area (MM-PBSA) methodology to estimate protein-protein binding energies. Protein figures were created using the freely available academic version of Maestro 9.7 Molecular Modeling Environment^48^.

### Stopped-flow fluorescence experiments

Proteins and their mutants were prepared as detailed in our previous article^4^. Kinetics of the binding was followed by measuring changes in the polarized AANAT-GFP fusion protein fluorescence intensity. Experiments were performed in a Hi-Tech Scientific PQ/SF-53 spectrofluorometer (dead time 0.7 ns) equipped with a high-intensity xenon arc lamp. The reaction was made in 0.1 M Hepes at pH 6.8 and 8 °C.

## Additional Information

**How to cite this article:** Masone, D. *et al*. On the role of residue phosphorylation in 14-3-3 partners: AANAT as a case study. *Sci. Rep.* **7**, 46114; doi: 10.1038/srep46114 (2017).

**Publisher's note:** Springer Nature remains neutral with regard to jurisdictional claims in published maps and institutional affiliations.

## Supplementary Material

Supplementary Information

## Figures and Tables

**Table 1:** 14-3-3 and AANAT residues involved in restrained dynamics for final near-native complex formation.

14-3-3	AANAT	D[Å]
Glu39	Gln132	10.7
Gln15	Arg142	8.95
Gln219	Glu43	7.86
Asn42	Asn35	6.54
Leu216	Arg38	7.06
Arg56	Thr31	10.2
