# Supplementary material for: On the role of residue phosphorylation in 14-3-3 partners: AANAT as a case study
Source: Sci Rep. 2017 Apr 7;7:46114. doi: 10.1038/srep46114 (PMC5384239; doi:10.1038/srep46114)
Supplement: Supplementary Information [file srep46114-s1.pdf]

Article title: “On the role of residue phosphorylation in 14-3-3 partners: AANAT as a case study”

Authors:
Diego Masone [1,2]; Marina Uhart[1] and Diego M. Bustos [1,3,*]

Affillations:
1 Instituto de Histología y Embriología (IHEM) - Consejo Nacional de Investigaciones Científicas y Técnicas (CONICET), CC56, Universidad Nacional de Cuyo (UNCuyo), 5500, Mendoza, Argentina.
2 Facultad de Ingeniería, Universidad Nacional de Cuyo (UNCuyo), Mendoza, Argentina.
3 Facultad de Ciencias Exactas y Naturales, Universidad Nacional de Cuyo (UNCuyo), Mendoza, Argentina.
* dbustos@mendoza-conicet.gob.ar

Supplementary information

Legend

Results of the three independent simulations of full molecular dynamics process from initial structures, through pseudo-native to native complex.
a) Glu87 and Arg89 distance to groups 1 and 2 in 14-3-3ζ (see the methods section).
The asterisk means simulation was performed with Thr31 in AANAT in its phosphorylated state
b) Normalized solvent accessible surface area (SASA) of the full protein-protein complex.
c) RMSD Cα to native structure. Sections marked as I, II or III in the plots correspond to first harmonic potential applied (I), free simulation (II) and second harmonic potential applied (III)

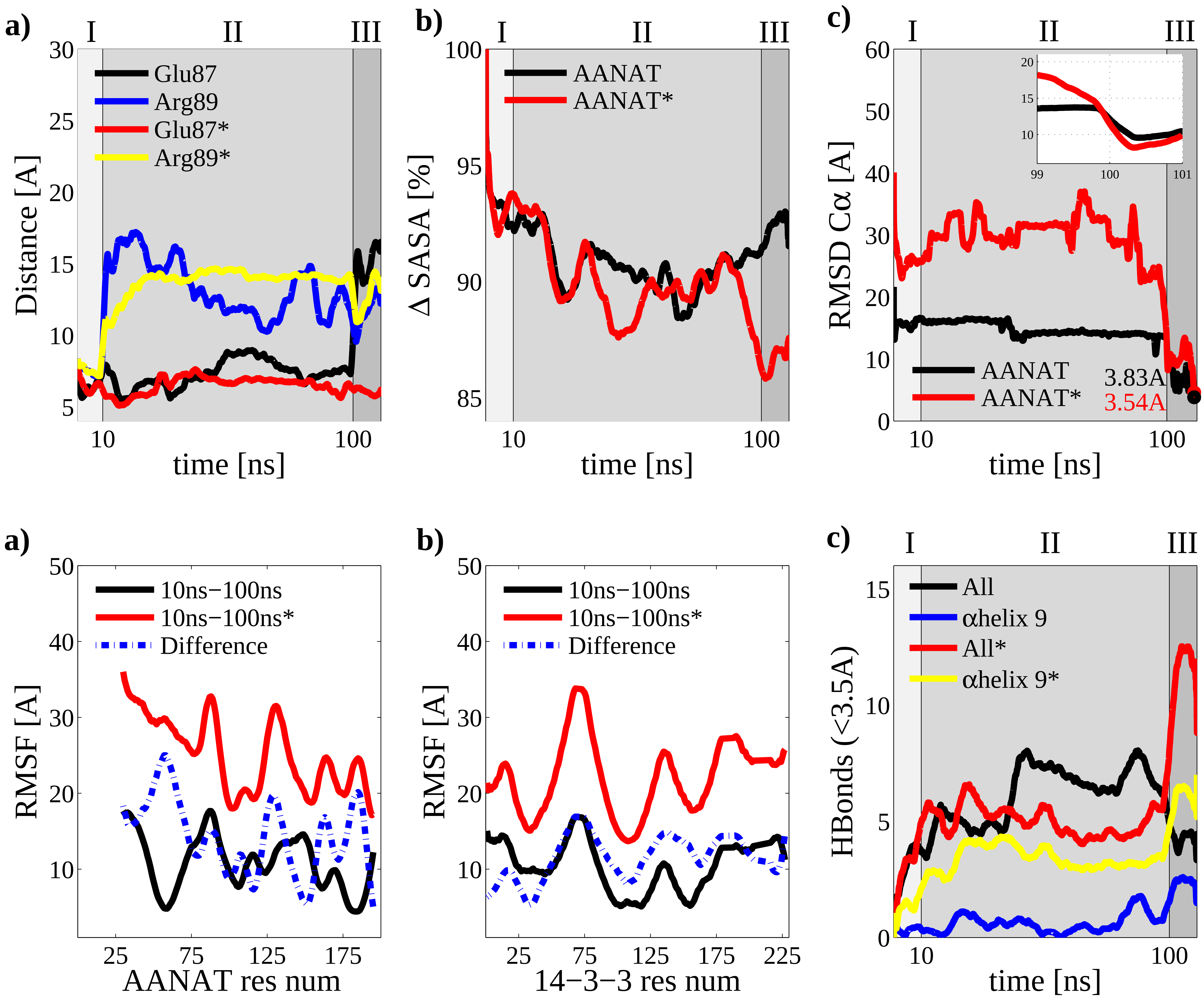

a)
I
II
III
Glu87
Arg89
Glu87*
Arg89*
Distance [A]
time [ns]
b)
AANAT
AANAT*
Δ SASA [%]
time [ns]
c)
AANAT
AANAT*
3.83A
3.54A
RMSD Cα [A]
time [ns]
a)
10ns−100ns
10ns−100ns*
Difference
RMSF [A]
AANAT res num
b)
10ns−100ns
10ns−100ns*
Difference
RMSF [A]
14−3−3 res num
c)
All
αhelix 9
All*
αhelix 9*
HBonds (<3.5A)
time [ns]

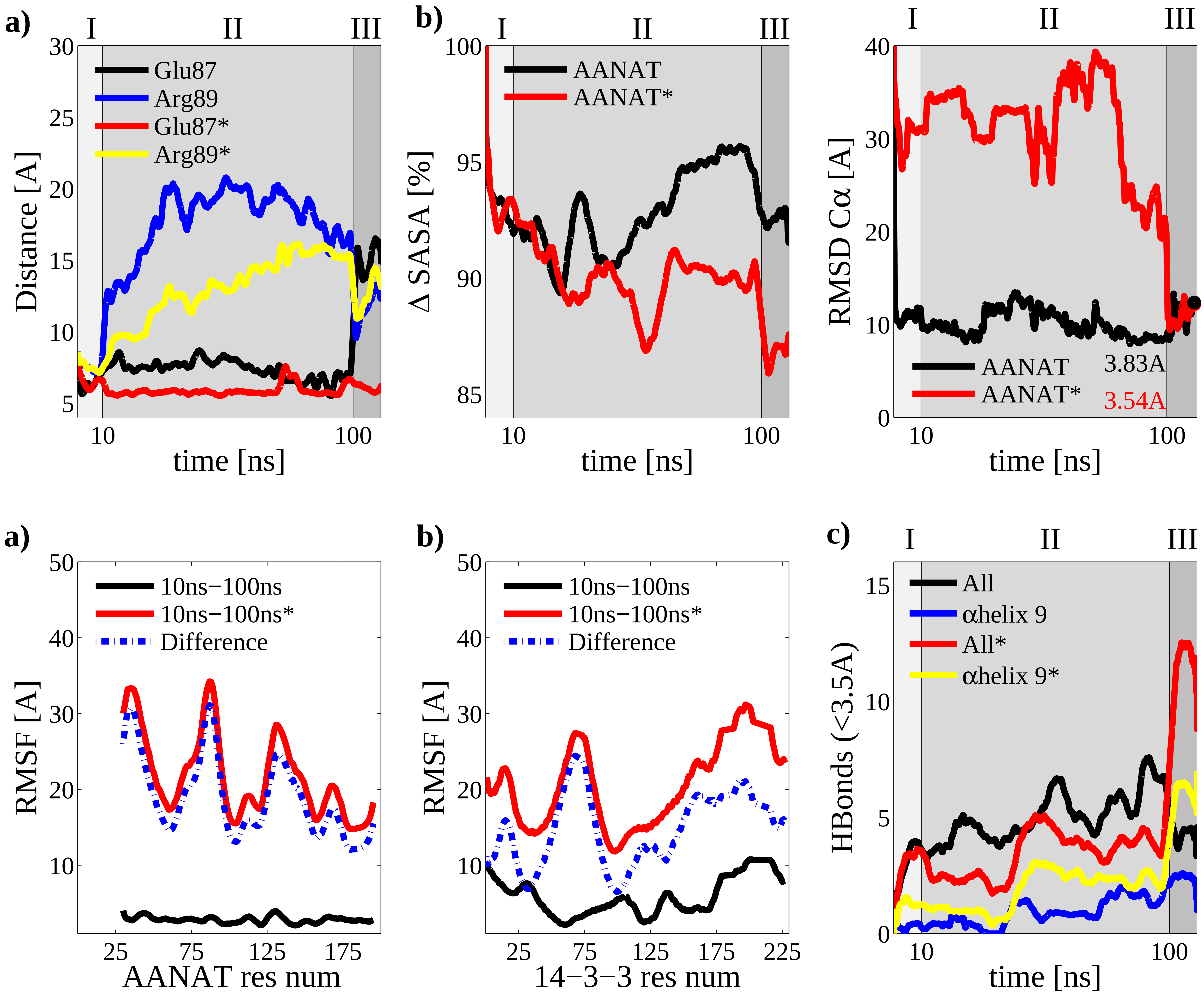

a)
I
II
III
Glu87
Arg89
Glu87*
Arg89*
Distance [A]
time [ns]
b)
AANAT
AANAT*
Δ SASA [%]
time [ns]
RMSD Cα [A]
3.83A
3.54A
time [ns]
a)
10ns−100ns
10ns−100ns*
Difference
RMSF [A]
AANAT res num
b)
RMSF [A]
14−3−3 res num
c)
All
αhelix 9
All*
αhelix 9*
HBonds (<3.5A)
time [ns]

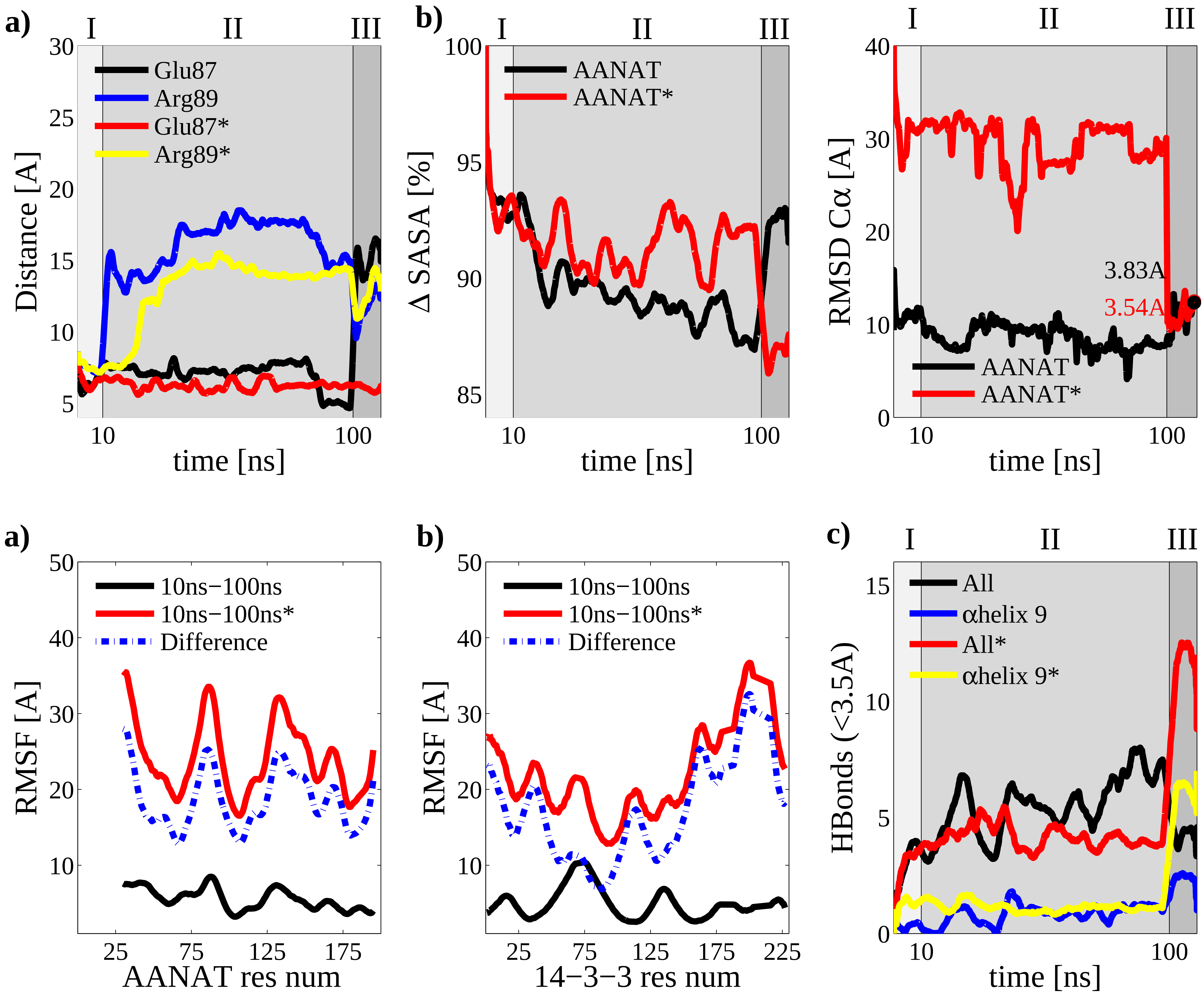
a)
I
II
III
Glu87
Arg89
Glu87*
Arg89*
Distance [A]
time [ns]
b)
AANAT
AANAT*
Δ SASA [%]
time [ns]
RMSD Cα [A]
3.83A
3.54A
AANAT
AANAT*
time [ns]
a)
10ns−100ns
10ns−100ns*
Difference
RMSF [A]
AANAT res num
b)
10ns−100ns
10ns−100ns*
Difference
RMSF [A]
14−3−3 res num
c)
All
αhelix 9
All*
αhelix 9*
HBonds (<3.5A)
time [ns]
